# Prenylated isoflavonoids from Fabaceae against the NorA efflux pump in *Staphylococcus aureus*

**DOI:** 10.1038/s41598-023-48992-8

**Published:** 2023-12-18

**Authors:** Marina Ika Irianti, Jean-Paul Vincken, Sarah van Dinteren, Ellen ter Beest, Klaas Martinus Pos, Carla Araya-Cloutier

**Affiliations:** 1 Laboratory of Food Chemistry, Wageningen University and Research, Bornse Weilanden 9, 6708 WG Wageningen, The Netherlands; 2 Laboratory of Microbiology and Biotechnology, Faculty of Pharmacy, Universitas Indonesia, Depok, 16424 Indonesia; 3 Institute of Biochemistry, Goethe-University Frankfurt, 60438 Frankfurt am Main, Germany

**Keywords:** Microbiology, Plant sciences, Drug discovery, Infectious diseases, Bacterial infection

## Abstract

Overexpression of NorA efflux pumps plays a pivotal role in the multidrug-resistance mechanism in *S. aureus*. Here, we investigated the activities of prenylated isoflavonoids, present in the legume plant family (Fabaceae), as natural efflux pump inhibitors (EPIs) in fluoroquinolone-resistant *S. aureus*. We found that four prenylated isoflavonoids, namely neobavaisoflavone, glabrene, glyceollin I, and glyceollin III, showed efflux pump inhibition in the *norA *overexpressing *S. aureus*. At sub-inhibitory concentrations, neobavaisoflavone (6.25 µg/mL, 19 µM) and glabrene (12.5 µg/mL, 39 µM), showed up to 6 times more Eth accumulation in *norA* overexpressing *S. aureus* than in the control. In addition, these two compounds boosted the MIC of fluoroquinolones up to eightfold. No fluoroquinolone potentiation was observed with these isoflavonoids in the *norA* knockout strain, indicating NorA as the main target of these potential EPIs. In comparison to the reported NorA EPI reserpine, neobavaisoflavone showed similar potentiation of fluoroquinolone activity at 10 µM, higher Eth accumulation, and less cytotoxicity. Neobavaisoflavone and glabrene did not exhibit membrane permeabilization effects or cytotoxicity on Caco-2 cells. In conclusion, our findings suggest that the prenylated isoflavonoids neobavaisoflavone and glabrene are promising phytochemicals that could be developed as antimicrobials and resistance-modifying agents to treat fluoroquinolone-resistant *S. aureus* strains.

## Introduction

Antimicrobial resistance (AMR) has become a fundamental problem worldwide. Recently, the COVID-19 pandemic accelerated the increase of resistance due to unnecessary antimicrobial use in hospitals^1^. Discovering new antibiotics remains a challenge and among numerous antibiotic candidates, only a few compounds have passed clinical trials in the last decade, including Zemdri (aminoglycoside), Xerava (tetracycline), and Nuzyra (tetracycline)^2^. To combat the rapid emergence of AMR, the development of novel therapeutic strategies, either with new antibiotics such as teixobactin or with resistance-modifying agents (RMAs), is urgently needed. RMAs are compounds that potentiate antimicrobial activity by perturbing multidrug resistance mechanisms^3,4^. The use of RMAs in drug combination therapy is gaining more interest because of their increased efficacy to tackle AMR^5^.

*Staphylococcus aureus* is one of the most widespread pathogens not only in hospital and community settings but also in livestock farming^6,7^. *Staphylococcus aureus* can develop resistance against several antimicrobials including vancomycin, fluoroquinolones, β-lactams, and the latest oxazolidinones (e.g., linezolid)^8–11^. Extensive investigations have been conducted to study putative mechanisms underlying resistance in *S. aureus*, such as the mutation of *gyrA* in DNA gyrase and the overexpression of efflux pump genes^12,13^. Efflux pumps play a pivotal role in AMR by extruding a broad range of noxious compounds, such as antibiotics, dyes, biocides, and other organic molecules and contribute to intrinsic and acquired resistance of bacteria^14^. Among different efflux pumps, members of the Major Facilitator Superfamily (MFS) are the most prevalent efflux pumps in gram-positive bacteria, including in *S. aureus*, for which the overexpression of NorA MFS efflux pump is observed in 43% of strains, particularly in MRSA strains^15^.

Considering their important role in the emergence of AMR, NorA efflux pumps are an interesting target for the development of RMAs. The use of EPIs can re-sensitize bacteria resistant to antibiotics^16^. Moreover, several EPIs are involved in impeding the evolution of resistance genes and decreasing the biofilm formation in *S. aureus*^17–19^. Unfortunately, there has been very little success in developing EPI as RMAs for clinical use. Whereas several synthetic EPIs have shown potent inhibition of efflux pump activity, they often suffer from off-target effects, cell toxicity and solubility issues^20^.

Plants can produce a myriad of metabolites as a response to microbial stress, which can be used as antimicrobials or RMAs. Various plant-derived compounds have been reported as NorA EPIs working synergistically with antibiotics, such as reserpine, 5′-methoxy-hydnocarpin (5′-MHC), totarol, epicatechin gallate and epigallocatechin gallate^21–24^. Amongst the NorA EPIs are some isoflavones namely biochanin A and genistein, other reported NorA EPIs are orobol, prenylated coumarin osthole and the prenylated flavonoid sophoraflavanone G^25–27^. Prenylated isoflavonoids are mainly found in the plant family Fabaceae^28^. These compounds are interesting sources for novel antimicrobials due to their diverse antimicrobial mechanisms, including membrane disruption, inhibition of ATP synthesis, reduction of biofilm formation, and inhibition of efflux pumps^29^. Prenylated isoflavonoids are substituted with hydrophobic prenyl (five-carbon isoprenoid) groups that exist in different configurations in nature (e.g. pyran, furan or chain prenyl)^30^. Prenylation of isoflavonoids usually occurs as a plant’s defense response to stress against both biotic (e.g., pathogenic microorganisms) or abiotic (e.g., oxidative stress) conditions^31,32^.

**Figure 1:**
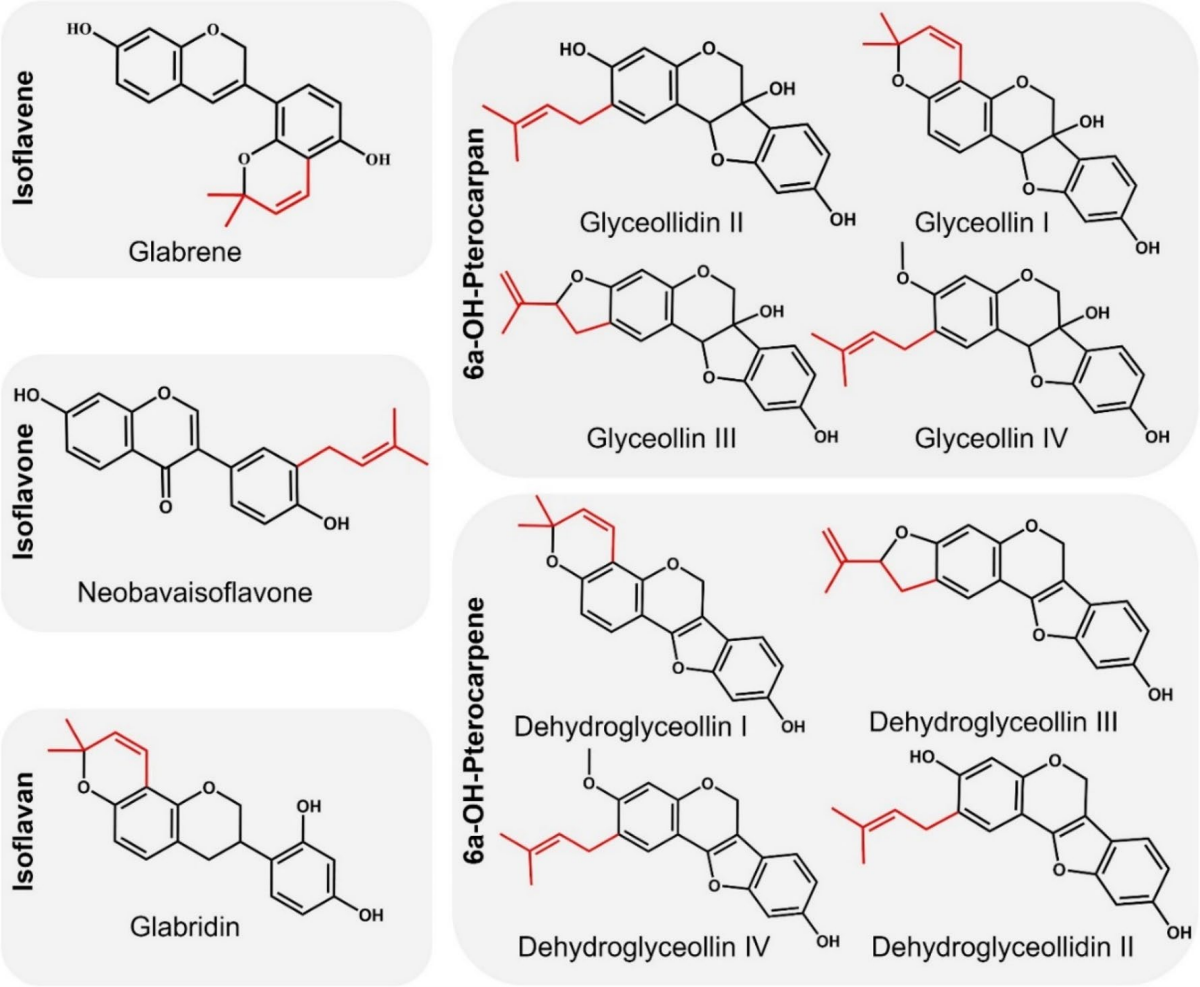
Overview of prenylated isoflavonoids of different subclasses, tested in this study. Prenyl groups are highlighted in red.

Here, we investigated prenylated isoflavonoids of 5 different subclasses and with different prenyl configurations (Fig. 1). The aim of this research was to evaluate whether prenylated isoflavonoids are effective against fluoroquinolone-resistant *S. aureus*, either as antimicrobials and/or as NorA EPIs. We hypothesize that prenylated isoflavonoids will have a high chance of acting as EPI since their hydrophobic features facilitate the penetration of compounds into the bacterial cell membrane and the binding of compounds into the binding pocket of the efflux pumps^33^. Moreover, these hydrophobic molecules possibly act as antimicrobials by disrupting bacterial membrane integrity^34^. This study aims to identify potential natural EPIs which might be studied further as potential RMAs against resistant *S. aureus*.

## Results

### Screening of prenylated isoflavonoids as potential NorA inhibitors

**Figure 2:**
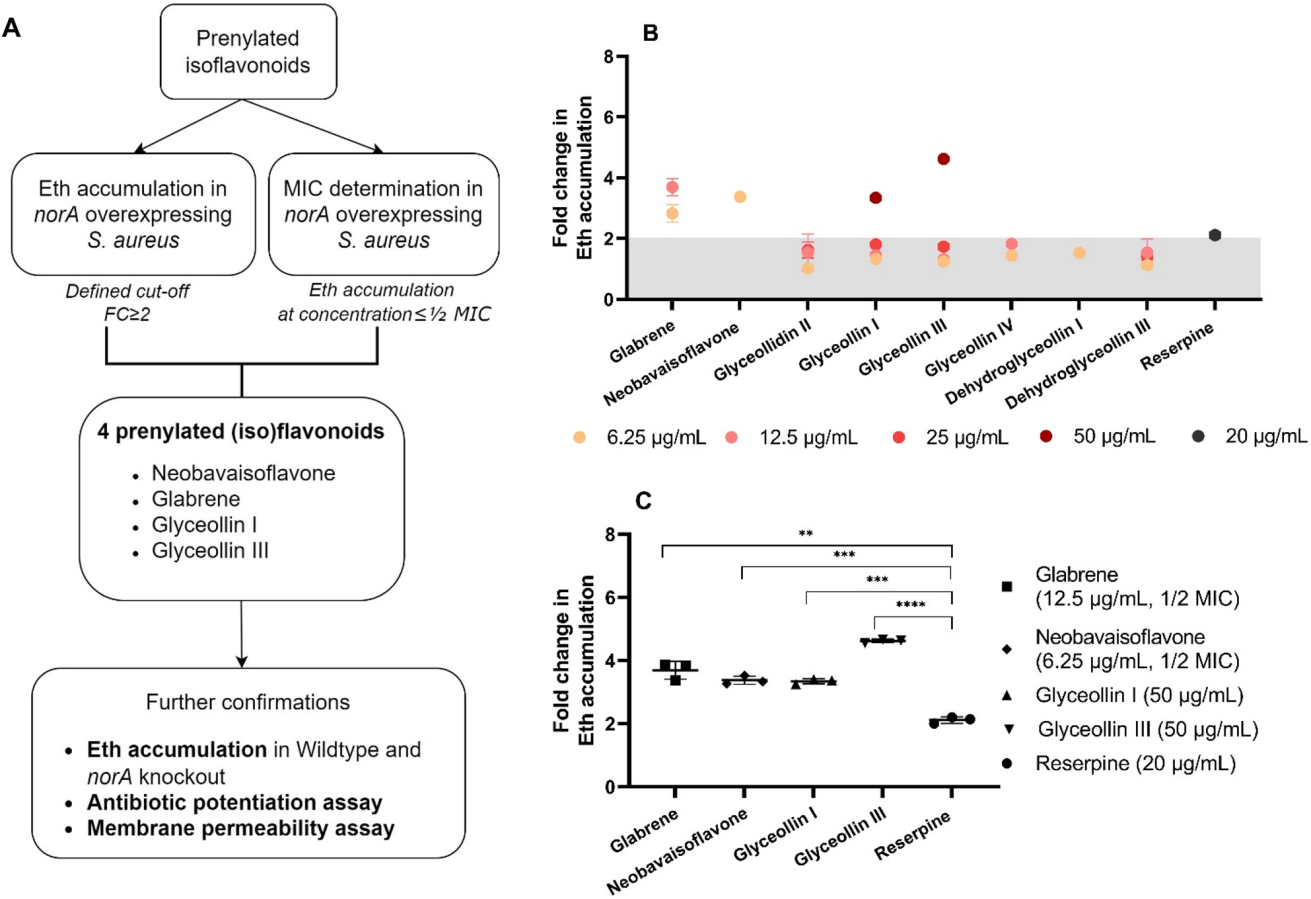
(**A**) Schematic diagram of the screening of prenylated isoflavonoids as candidate NorA EPIs; (**B**) Eth accumulation results with 11 tested compounds in the *norA* overexpressing strain (SA-1199B*)*. The grey area corresponds to ≤ twofold change of fluorescence intensity (lower than positive control with reserpine); The dots represent the fold change of Eth accumulation at sub-inhibitory concentrations. Glabridin, dehydroglyceollidin IV, and dehydroglyceollidin II showed fold change ≥ 2 at MIC, therefore the data were not plotted in the Fig. 2B; (**C**) Eth accumulation results of the indicated candidate compounds that were used for further assays. Data were shown as an average of fold changes in Eth accumulation with standard deviations. Double asterisks denote a significant difference of *p* < 0.01, triple and quadruple asterisks denote *p* < 0.001 and *p* < 0.0001, respectively. Statistical significance was calculated with student’s t-test and Welch’s correction^35^.

The first screening was performed to narrow down the selection of potential NorA efflux pump inhibitors (Fig. 2A). In this step, we used *norA* overexpressing strain to select the compounds that potentially act against NorA. EPI activity was plotted as a fold-change of Eth accumulation relative to its corresponding negative control. Intercalation of Eth to the intracellular DNA results in an enhanced quantum yield and therefore enhanced Eth fluorescence. SA-1199B overexpresses the *norA* efflux pump gene and shows an enhanced Eth efflux and consequently accumulates less dye, resulting in a low level of fluorescence. On the other hand, when EPIs block or competitively bind to NorA, Eth will not be effectively extruded, resulting in higher Eth accumulation and a higher fluorescence signal. Therefore, the fold changes in Eth accumulation correspond to the affinity of the compounds to bind and inhibit NorA resulting in lower efflux activity and higher Eth accumulation.

Of the 11 prenylated isoflavonoids tested in the Eth accumulation assay, we selected compounds that showed Eth accumulation higher than our positive control reserpine, an indole alkaloid compound and known NorA inhibitor, which showed a twofold fluorescence increase (Fig. 2B)^21^. Subsequently, antimicrobial activity was measured to exclude false-positive results in the Eth accumulation assay (detailed MIC values in Table S1). Based on our selection criteria (Fig. 2A), four compounds were considered potential EPIs, as they showed promising EPI activity at sub-inhibitory concentrations (up to ½ MIC). Those four compounds were chain-prenylated neobavaisoflavone, ring-prenylated glabrene, pterocarpans glyceollin I, and glyceollin III. Neobavaisoflavone, glabrene, glyceollin I, and glyceollin III reached nearly two times higher Eth accumulation than the positive control reserpine (Fig. 2C).

### Evaluating antimicrobial activities of prenylated isoflavonoids in *S. aureus*

Antimicrobial susceptibility assays were performed with neobavaisoflavone, glabrene, glyceollin I, glyceollin III, all four selected on basis of the results from the Eth accumulation assay (Fig. 2). We evaluated the MICs of the four compounds in the *S. aureus* wildtype strain SA-1199, the *norA* overexpressing strain SA-1199B and the *norA* knockout strain SA-K1758 (Table 1). The MIC values were examined to (1) characterize the activity of the candidate NorA EPIs against the other two strains of *S. aureus* and (2) check whether candidate NorA EPIs exerted antibacterial activities at the concentrations used in the Eth accumulation assay. Glabrene and neobavaisoflavone showed good antimicrobial activity with MIC values lower than 50 µg/mL in all strains. Neobavaisoflavone showed MIC and MBC values of 12.5 µg/mL and 25 µg/mL, respectively, in all *S. aureus* strains. Glabrene showed good antimicrobial activity with a MIC of 12.5–25 µg/mL against the wildtype SA-1199 and knockout SA-K1758, and MIC of 25 µg/mL against the overexpressing SA-1199B. Glyceollin I and III were considered moderate or weak antimicrobials due to their high MICs (≥ 50 µg/mL). Among other tested control compounds, ciprofloxacin and norfloxacin showed the highest MIC in *norA* overexpressing strain SA-1199B and the lowest MIC in *norA* knockout strain SA-K1758, which corroborates that these fluoroquinolones are substrates of NorA. Unlike for the fluoroquinolones, the overexpression of *norA* did not remarkably affect the MIC of prenylated isoflavonoids. Erythromycin showed an MIC value of 0.5–1 µg/mL in the *norA* overexpressing strain SA-1199B and was non-antimicrobial in the *norA* knockout strain SA-K1758 due to the chromosomally located *erm* resistance cassette^23,36^. The dye Eth and the known NorA inhibitor reserpine were found non-antimicrobial up to 50 µg/mL.

**Table 1:** Overview of MIC and MBC of prenylated isoflavonoids, antibiotics, and other compounds against SA-1199, SA-1199B, and SA-K1758.

Subclass	Compound	SA-1199 (wildtype)		SA-1199B (*norA* overexpressing)		SA-K1758 (*norA* knockout)	
		MIC µg/mL [µM]	MBC µg/mL [µM]	MIC µg/mL [µM]	MBC µg/mL [µM]	MIC µg/mL [µM]	MBC µg/mL [µM]
Isoflavones	Neobavaisoflavone	12.5 [39]	25 [78]	12.5 [39]	25 [78]	12.5 [39]	25 [78]
Isoflavene	Glabrene	12.5 < MIC < 25 [39 < MIC < 78]	25 [78]	25 [78]	50 [155]	12.5 < MIC < 25 [39 < MIC < 78]	25 [78]
Pterocarpans	Glyceollin I	80	ND^a^	80 [236]	ND^a^	60 [177]	80 [236]
	Glyceollin III	> 50 [> 148]	ND^a^	80 [236]	ND^a^	50 [148]	ND^a^
Others	Reserpine	> 50 [> 82]	ND^a^	> 50 [> 82]	ND^a^	> 50 [> 82]	ND^a^
	Ciprofloxacin	0.25 [0.75]	0.5 [1.5]	8 [24]	16 [48]	0.125 [0.38]	0.25 [1.5]
	Norfloxacin	ND^a^	ND^a^	45 [141]	ND^a^	0.2 [0.63]	0.25 [0.78]
	Erythromycin	ND^a^	ND^a^	0.5–1 [0.7–1.4]	ND^a^	> 100 [> 136]	> 100 [> 136]
	EtBr	> 50 [> 127]	ND^a^	> 50 [> 127]	ND^a^	> 50 [> 127]	ND^a^

The antimicrobial activities were tested in technical triplicates with at least two biological repetitions each. ^a^ND, not determined.

### Evaluating efflux pump inhibition by prenylated isoflavonoids

**Figure 3:**
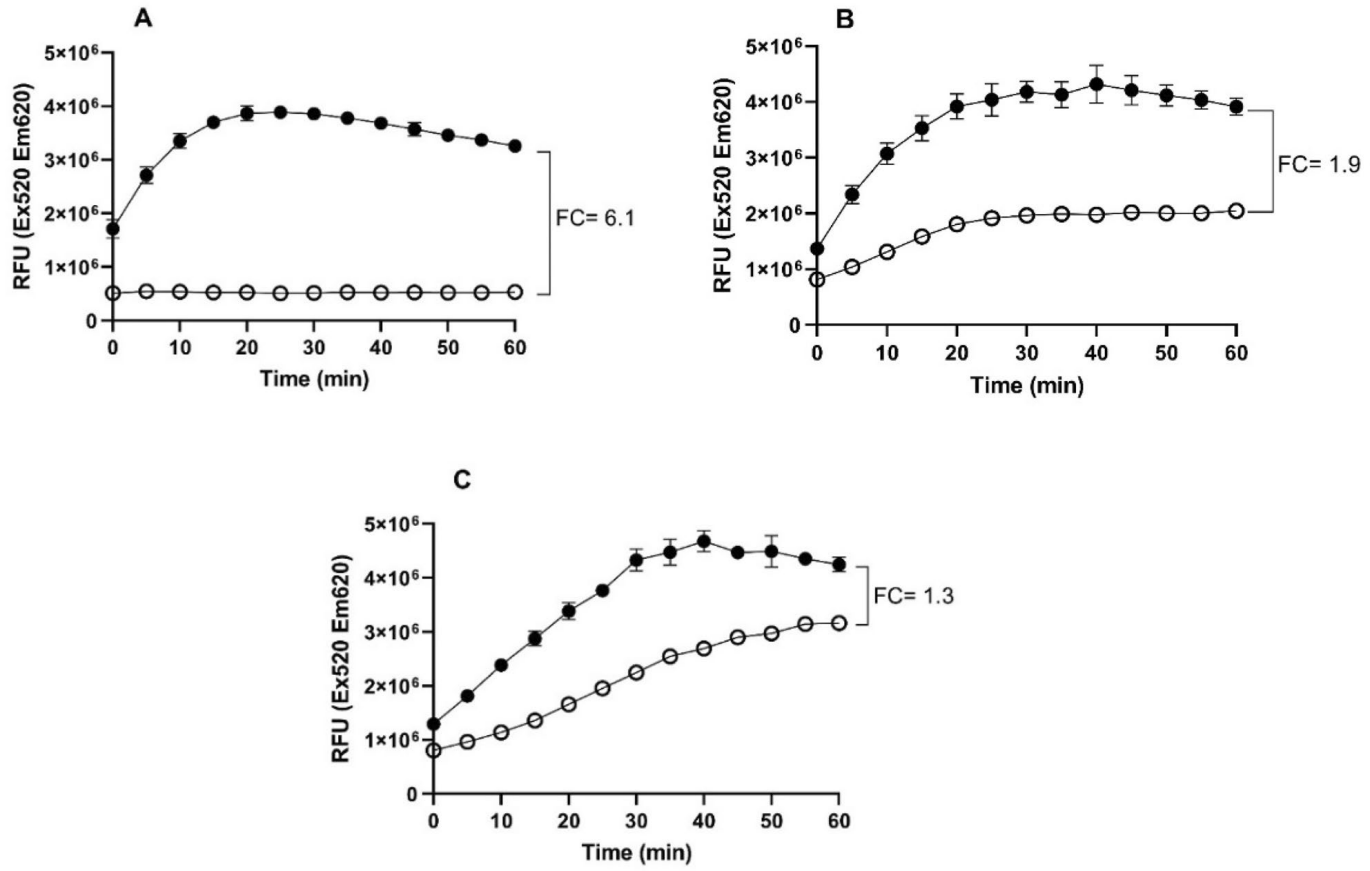
Eth accumulation in the presence of glabrene at a concentration of 6.25 µg/mL (1/4 MIC) in (**A**) *norA* overexpressing strain (SA-1199B), (**B**) wildtype strain (SA-1199), (**C**) *norA* knockout strain (SA-K1758). The open circles correspond to the control (cells without glabrene) and the closed (black) circles correspond to cells with glabrene. In these graphs, representative data from three technical replicates of two biological experiments is shown. FC is a fold change in the increase of Eth accumulation after 60 min measurement. The average of two biological repetitions in Eth accumulation graphs were presented in the supplementary Fig. S1 to S3.

To validate the specificity of prenylated isoflavonoids as NorA EPIs, we executed time-resolved Eth accumulation assays in the presence and absence of glabrene with the wildtype SA-1199, *norA* knockout strain SA-K1758, and the *norA* overexpressing strain SA-1199B (Fig. 3). For the wildtype and *norA* knockout strain, Eth is gradually accumulated during time even in the absence of glabrene, whereas the *norA* overexpressing strain does not show accumulation of Eth, indicating a strong Eth efflux activity. In the presence of glabrene (at ¼ of the MIC value), an enhanced accumulation rate and final Eth accumulation to approx. sixfold increase is shown. The same steady state level is observed for all three strains. The fold changes of the Eth accumulation after 60 min in the presence vs. the absence of glabrene is 6.1, 1.9, and 1.3 for the *norA* overexpressing, wildtype or *norA* knockout strain, respectively.

**Figure 4:**
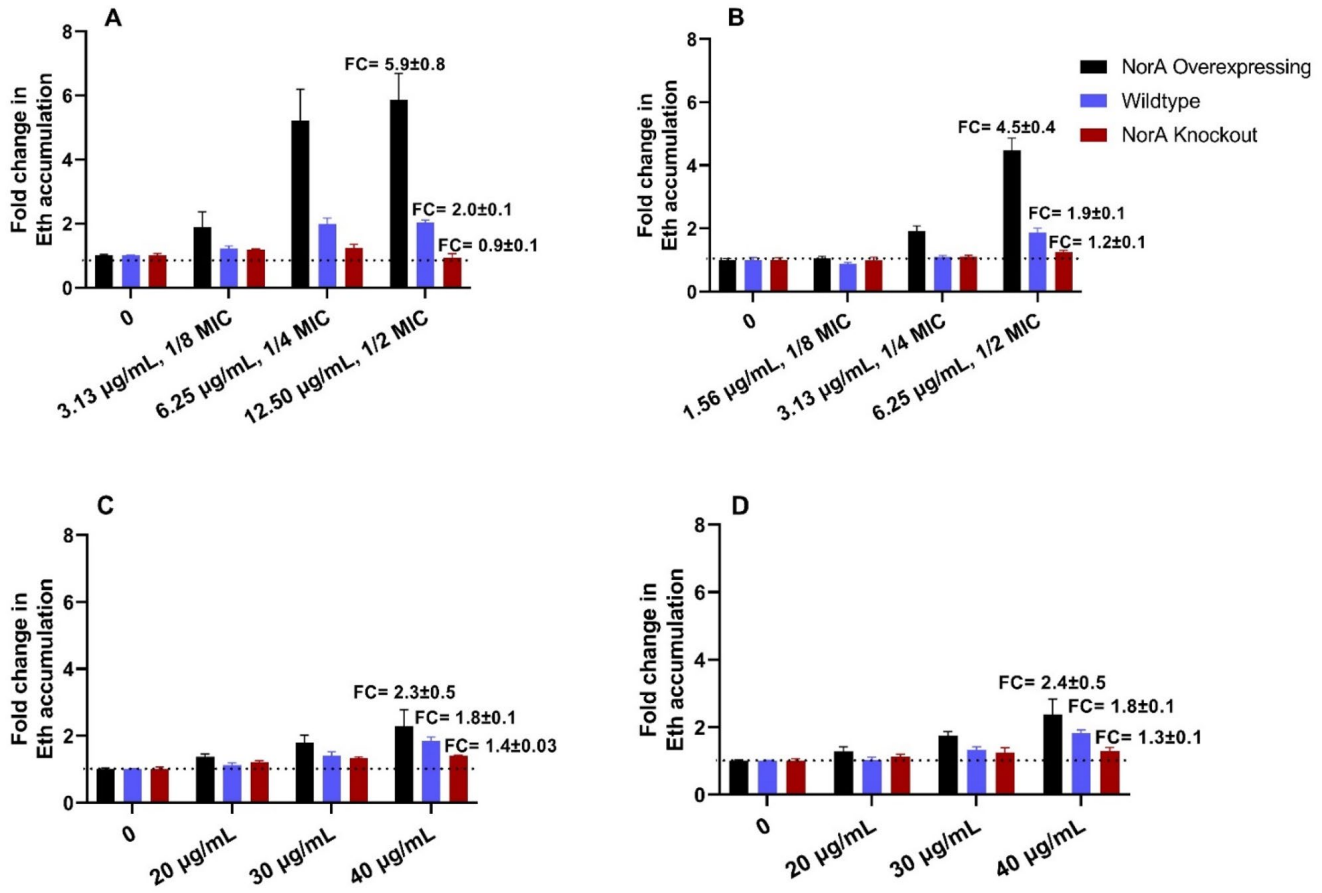
Effect of different concentrations of prenylated isoflavonoids (**A**) glabrene, (**B**) neobavaisoflavone, (**C**) glyceollin I, (**D**) glyceollin III, on the fold change (FC) in Eth accumulation after 1 h measurement with *norA* overexpressing (SA-1199B), wildtype (SA-1199), and *norA* knockout (SA-K1758) strains. The glabrene and neobavaisoflavone concentrations used in Fig. 4A and B were chosen based on MIC values in SA-1199B (MIC glabrene 25 µg/mL; MIC neobavaisoflavone 12.5 µg/mL, MIC glyceollin I and glyceollin III > 50 µg/mL).

Eth accumulation experiments were also conducted in the absence and presence of neobavaisoflavone, glyceollin I and glyceollin III (Fig. S1 to S3). The compounds alone (no cells) in buffer with and without EtBr did not emit fluorescence, as shown in Fig. S4. In Fig. 4, the fold change (FC) of Eth accumulation after 60 min in the presence vs. the absence of the prenylated isoflavonoids for each of the three different *S. aureus* strains is shown. For all prenylated isoflavonoids, we found a notable FC of Eth accumulation with the *norA* overexpressing strain, and smaller FCs in the wildtype and the *norA* knockout strain (fold changes in SA-1199B > SA-1199 > SA-K1758). At the maximum concentration tested in *norA* overexpressing strain SA-1199B, glabrene showed the highest fold change in Eth accumulation (FC = 5.9 ± 0.8), followed by neobavaisoflavone (FC = 4.5 ± 0.4). Glyceollin I and glyceollin III also displayed higher activity in the *norA* overexpressing strain with FCs of 2.3 ± 0.5 and 2.4 ± 0.5, respectively. These results denote that NorA is potentially the major target of the tested compounds, particularly glabrene and neobavaisoflavone, suggesting them as the most promising EPI candidates. However, since minor FCs between 0.9 and 1.4 were observed for the *norA* knockout strain SA-K1758, NorA is potentially not the only efflux pump affected by the tested compounds or the compounds affect the membrane permeability in the *norA* knockout strain. We therefore performed further assays, such as antibiotic potentiation with NorA substrates and non-NorA substrates and membrane permeabilization assays to address the NorA-specificity of the prenylated isoflavonoids.

### Evaluating antibiotic potentiation effect of prenylated isoflavonoids

Efflux of fluoroquinolones by NorA has been shown to be an effective resistance mechanism against these antibiotics^37^. To verify whether prenylated isoflavonoids modulate the antibacterial activity of fluoroquinolones, we performed checkerboard assays in both *norA* overexpressing and *norA* knockout strains. Table 2 displays the minimal inhibitory concentration (MIC) of ciprofloxacin, norfloxacin, and erythromycin in absence and presence of prenylated isoflavonoids. Antibiotic potentiation is often described as fold reduction; the extent of the reduction of the MIC indicates the potency of compounds in modulating the efficacy of the antibiotics. We found that glabrene, neobavaisoflavone, glyceollin I, and glyceollin III were able to reduce the MIC of fluoroquinolones in the *norA* overexpressing strain SA-1199B by 4- to eightfold. When we standardized the antibiotic potentiation at fourfold reduction (to compare with the maximum fold reduction observed with the positive control reserpine, bold rows in Table 2), glabrene and neobavaisoflavone showed synergism with the tested fluoroquinolone antibiotics (FICI = 0.5). All prenylated isoflavonoids reduced the MIC of ciprofloxacin and norfloxacin fourfold (except for norfloxacin in combination with glabrene), matching the fold reduction inflicted by reserpine (Table 2). The modulators were used at different concentrations to elicit this effect, neobavaisoflavone was used at a concentration of 3.125 µg/mL (10 µM), glabrene at a concentration of 6.25 µg/mL (19 µM), and reserpine at a concentration of 6.25 µg/mL (10 µM). There was a slight potentiation by twofold observed in *norA* knockout strain SA-K1758 at the highest concentrations tested for glabrene and neobavaisoflavone. The twofold reduction in the *norA* knockout could be attributed to the more susceptible properties of the *norA* knockout towards antibiotics compared to the *norA* overexpressing strain. Moreover, both prenylated isoflavonoids and antibiotics can give an additive effect when applied at ½ the MIC values each, resulting in twofold reduction. The FICI values showed that glabrene acted in synergy (FICI = 0.5) with ciprofloxacin and neobavaisoflavone synergized with both fluoroquinolone antibiotics. Based on the fourfold reduction at 10 µM and apparent synergism with both fluoroquinolones, neobavaisoflavone showed the most promising activities among other tested prenylated isoflavonoids as a NorA EPI, in addition to its antimicrobial properties.

**Table 2:** MIC of ciprofloxacin, norfloxacin and erythromycin in combination with prenylated isoflavonoids for *S. aureus* SA-1199B and SA-K1758.

Compound	EPI conc. (µg/mL)	EPI conc.(µM)	SA-1199B						SA-K1758					
			MIC_CIP_^a^ [FR^b^]	FICI	MIC_NOR_^a^ [FR^b^]	FICI	MIC_ERY_^a^ [FR^b^]	FICI	MIC_CIP_^a^ [FR^b^]	FICI	MIC_NOR_^a^ [FR^b^]	FICI	MIC_ERY_^a^ [FR^b^]	FICI
Antibiotic (AB)			8		45		0.5–1		0.125		0.2		> 100	
AB + Glabrene	12.5 ( ½ MIC)	39	1 [8]	0.625	5.625 [8]	0.625	0.5[2]	1	0.063 [2]	1	–	–	ND^c^	–
	**6.25 ( 1/4 MIC)**	**19**	**2 [4]**	**0.50**	22.5 [2]	0.75	0.5[2]/-^d^	0.75/-	–	**–**	**–**	**–**	ND^c^	**–**
	3.125 ( 1/8 MIC)	10	4 [2]	0.625	–	–	–	–	–	–	–	–	ND^c^	–
AB + Neobavaisoflavone	6.25 ( ½ MIC)	19	1 [8]	0.625	5.625 [8]	0.625	0.5[2]	1	0.063 [2]	1	0.1 [2]	1	ND^c^	–
	**3.125(1/4 MIC)**	**10**	**2 [4]**	**0.50**	**11.25 [4]**	**0.5**	0.5[2]/–^d^	0.75/–	–	**–**	–	–	ND^c^	–
	1.563 ( 1/8 MIC)	5	4 [2]	0.625	22.5 [2]	0.625	0.5[2]/–^d^	0.63/–	–	–	–	–	ND^c^	–
AB + Glyceollin I	50	148	2 [4]	< 1.25	ND^c^	ND^c^	ND^c^	ND^c^	0.063 [2]	1.33	ND^c^	ND^c^	ND^c^	–
	**40**	**118**	**2 [4]**	** < 1.05**	**11.25 [4]**	**0.75**	0.5[2]/–^d^	1/–	**–**	**–**	–	–	ND^c^	–
	30	89	4 [2]	< 1.10	22.5 [2]	0.875	0.5[2]/–^d^	0.88/–	–	–	–	–	ND^c^	–
	20	59	4 [2]	< 0.90	22.5 [2]	0.75	0.5[2]/–^d^	0.75/–	–	–	–	–	ND^c^	–
AB + Glyceollin III	50	148	1 [8]	< 1.13	5.625 [8]	< 1.125	ND^c^	ND^c^	ND^c^	ND^c^	ND^c^	ND^c^	ND^c^	–
	40	118	2 [4]	< 1.05	11.25 [4]	< 1.05	0.25[2]/–^d^	–	0.063 [2]	1.30	0.1 [2]	–	ND^c^	–
	**30**	**89**	**2 [4]**	** < 0.85**	**11.25 [4]**	** < 0.85**	0.25[2]/–^d^	–	0.063 [2]	**1**	–	–	ND^c^	–
	20	59	4 [2]	< 0.90	22.5 [2]	< 0.9	–	–	–	–	–	–	ND^c^	–
AB + Reserpine	**6.25**	**10**	**4[2]/2[4]**^d^	< 0.625 / < **0.375**	**22.5 [2] /11.25 [4]**^d^	< 0.625 / < **0.375**	–	–	–	–	–	–	–	–

^a^MIC_**CIP**_, the MIC of ciprofloxacin (µg/mL); ^a^MIC_NOR_, the MIC of norfloxacin (µg/mL); ^a^MIC_ERY,_ the MIC of erythromycin, in combination with EPI or without EPI. ^b^FR, fold reduction. ^c^ND, not determined. “–” means no change in MIC of antibiotic was found and therefore also no fold reduction (FR) nor fractional inhibitory concentration index (FICI). FICI ≤ 0.5 synergy (indicated in bold); 0.5 < FICI < 1 partial synergy; FICI = 1 additive; 1 < FICI < 4 indifference and FICI ≥ 4.0 antagonism. If the compound had no determinable MIC value, the FICI was calculated with the highest concentration tested (50 µg/mL).^d^Results from the biological duplicates differed.

Pterocarpans glyceollin I and III showed a fourfold reduction of MICs of both fluoroquinolone antibiotics albeit at higher concentrations (89–118 µM) compared to glabrene and neobavaisoflavone. Pterocarpans were considered partial potentiators (0.5 < FICI < 1) in SA-1199B, while the effect was only additive or indifferent in SA-K1758 (1 ≤ FICI < 4).

A minor antibiotic potentiation (maximum two-fold reduction) was observed for prenylated isoflavonoids and reserpine with the non-fluoroquinolone antibiotic erythromycin. We did not assess the potentiation of erythromycin in the *norA* knockout strain SA-K1758 because the introduction of *erm* cassette to this strain resulted in resistance towards erythromycin^23,36^. To conclude, neobavaisoflavone and to a lesser extent glabrene, stand as the most promising antibiotic potentiator among the tested compounds in the checkerboard assay, in which the potentiation effect might be due to the inhibition of NorA efflux pumps.

### Evaluating membrane permeabilization effect of prenylated isoflavonoids

**Figure 5:**
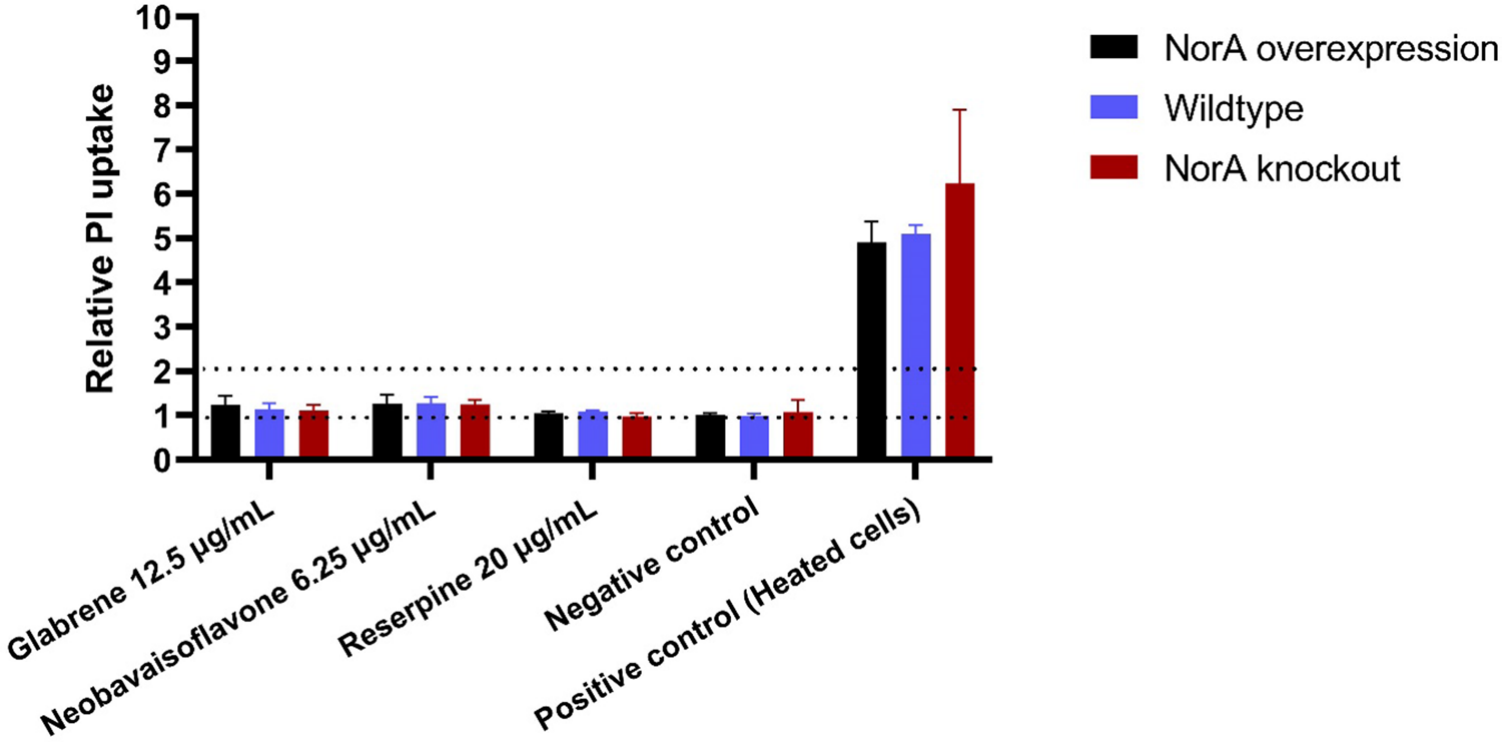
Effect of prenylated isoflavonoids and reserpine on membrane permeability at their maximum concentration tested in Eth accumulation assay. Data were expressed as relative propidium iodide (PI) uptake, which was obtained by taking final fluorescence after 2 h measurement and calculated relative to the negative control. The definitions of non-permeabilizers (relative PI uptake ≤ 1), poor permeabilizers (relative PI uptake 1–2), good permeabilizers (relative PI uptake > 2) are shown by dotted lines.

To check the membrane permeabilization effect of prenylated isoflavonoids, we conducted propidium iodide (PI) assays in the three *S. aureus* strains. PI is a fluorescent dye that produces high fluorescence when it binds to DNA. This dye is commonly used to assess the cell viability in mammalian cells, but also widely utilized to check bacterial membrane integrity^38,39^. When the cell membrane is not intact, like in heated cells, PI will enter the cells and produce an increase in fluorescence. According to Fig. 5, prenylated isoflavonoids showed some low levels of PI uptake at their maximum concentration tested in the Eth accumulation assay and antibiotic potentiation assay. We also performed the PI assay in the presence of the uncoupler carbonyl cyanide-m-chlorophenyl hydrazone (CCCP) affecting the proton motive force (PMF), as the extent of the PMF may influence propidium ion permeability^40^. As propidium has a similar structure in terms of the phenanthridine ring as Eth, which is one of the substrates for diverse efflux pumps, we wanted to confirm that the low PI uptake was not due to the extrusion of PI by the efflux pumps (after permeabilization of the membrane)^41,42^. By dissipating the PMF and therefore inactivating the efflux PMF (uncoupling the pumps), we were able to exclude propidium’s extrusion (Fig S5). Based on our results, prenylated isoflavonoids did not show permeabilization properties up to ½ MIC with and without CCCP in all *S. aureus* strains.

### Evaluating cytotoxicity and cell viability of prenylated isoflavonoids

**Figure 6:**
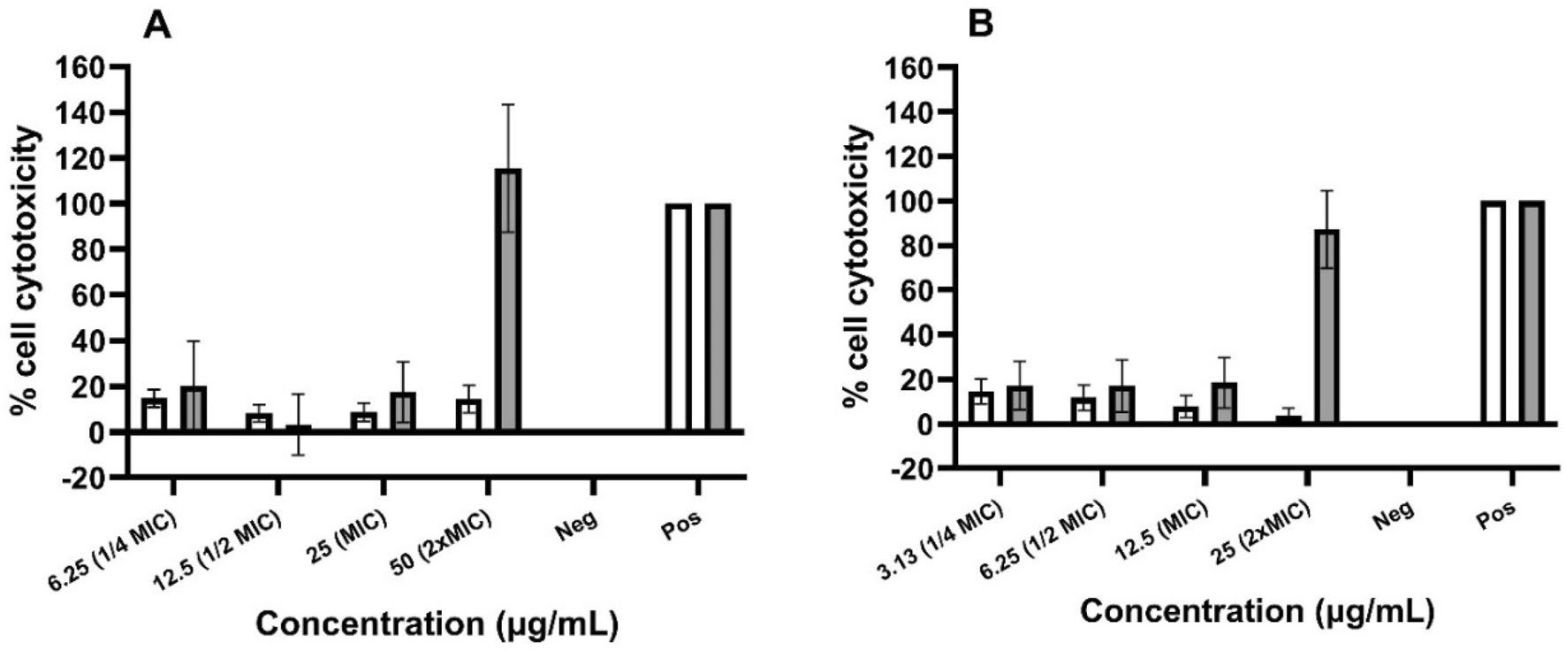
Cell cytotoxicity of (**A**) glabrene and (**B**) neobavaisoflavone as assayed via LDH measurement after 4 h (white) and 24 h (grey) incubation. Negative control (cells with medium) was set 0% and positive control (cells with 1% Triton X-100) was set 100%.

The cytotoxicity of glabrene and neobavaisoflavone was determined against Caco-2 cells. According to the LDH assay, these prenylated isoflavonoids did not exhibit profound cell cytotoxicity after 4 h and 24 h incubation, where the cell cytotoxicity was established (in comparison to the positive control) at concentrations up to 25 µg/mL (78 µM) and 12.5 µg/mL (39 µM), respectively (Fig. 6). In addition, no notable cytotoxicity effect was seen with glabrene and neobavaisoflavone after 4 h incubation at concentrations up to 50 µg/mL (155 µM) and 25 µg/mL (78 µM), respectively. However, cytotoxicity was observed after 24 h incubation at the same concentrations.

**Figure 7:**
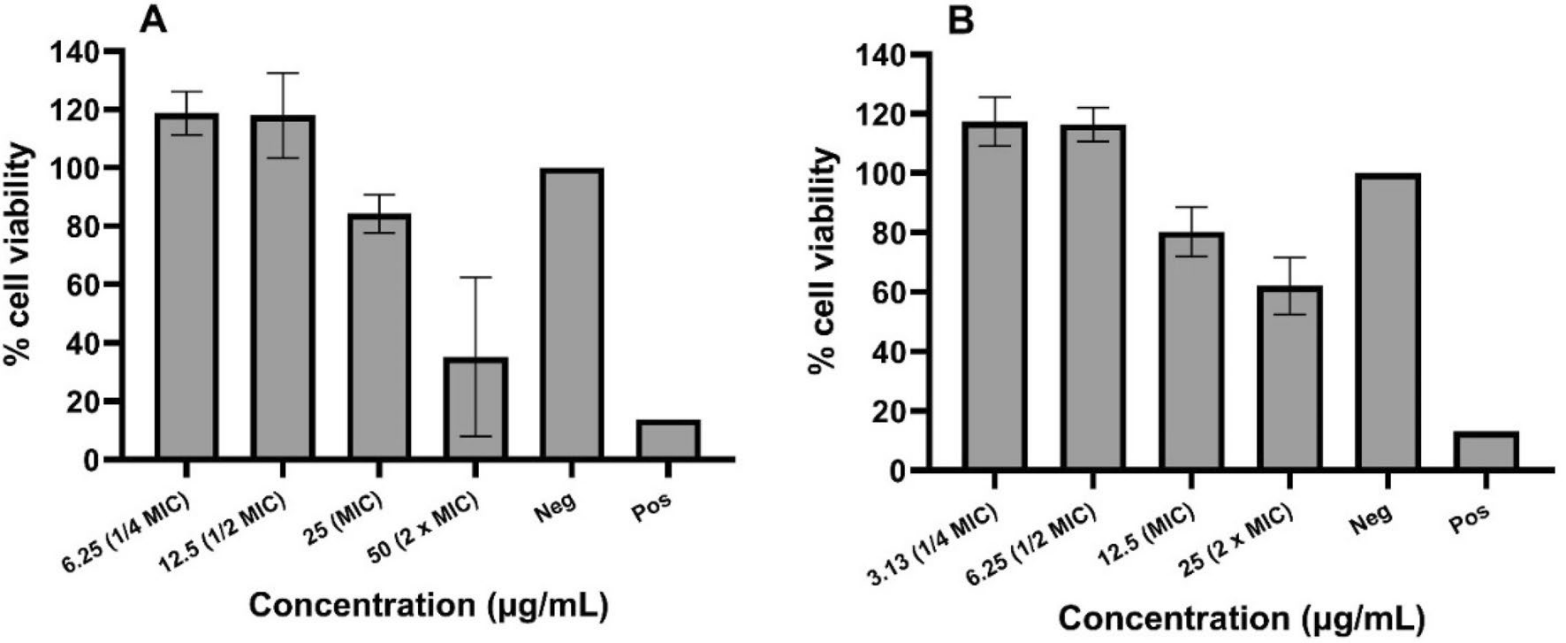
Cell viability assessment of Caco-2 cell lines in the presence of (**A**) glabrene and (**B**) neobavaisoflavone as assayed via WST-1 measurement after 24 h incubation. The negative control were cells with growth medium and the positive control were cells treated with 1% v/v Triton X-100.

We carried out a cell viability assay to further demonstrate the effect of the compounds on the metabolic activity of cells (Fig. 7). Glabrene and neobavaisoflavone did not affect cell viability up to ½ MIC, which corresponds to the LDH assay results (Fig. 6). However, the cell viability was slightly reduced to 80% at MIC and further reduction was observed at 2 × MIC. According to these findings, neither glabrene nor neobavaisoflavone had any discernible cytotoxic effects at the tested EPI concentrations.

## Discussion

### Prenylated isoflavonoids act as potential NorA efflux pump inhibitors in *S. aureus*

According to the criteria described by Lomovskaya et al. and Opperman et al*.*, prenylated isoflavonoids, particularly glabrene and neobavaisoflavone, seem to be promising EPI candidates based on (1) increased level of Eth accumulation in *norA* overexpressing strain, (2) antibiotic potentiation in *norA* overexpressing strain, (3) no significant antibiotic potentiation effect in *norA* knockout strain, (4) no remarkable antibiotic potentiation with an antibiotic which is not the main substrate for NorA^43,44^. To ensure the EPI activity towards NorA, we performed antibiotic potentiation assays by combining prenylated isoflavonoids with fluoroquinolones (substrates of NorA) and erythromycin (non-substrate of NorA). The lower potentiation effect that was found in the *norA* knockout and higher potentiation in the *norA* overexpressing strain indicates that the mode of action was associated with the NorA efflux pump. Our potentiation results with neobavaisoflavone are in line with those of Abreu et al*.*, where a FICI of 0.50 was observed with fourfold ciprofloxacin MIC reduction^45^. However, we found up to eightfold ciprofloxacin reduction at ½ MIC. Compared to Abreu et al*.,* we used a more comprehensive approach to confirm the activity of neobavaisoflavone as a NorA inhibitor by performing the assay with the *norA* knockout strain as well^45^. Glyceollin I and glyceollin III showed comparable inhibitory behavior, where fourfold to eightfold reduction of ciprofloxacin and norfloxacin MIC were observed in the *norA* overexpressing strain at concentrations up to 50 µg/mL. Yet, glyceollin I and glyceollin III required higher concentrations than glabrene and neobavaisoflavone. Furthermore, glyceollin I can only reduce the MIC of fluoroquinolones by a maximum of fourfold. We tested the potentiation effect of these four prenylated isoflavonoids with the non-fluoroquinolone antibiotic erythromycin to ensure the specificity of prenylated isoflavonoids as NorA EPI. Erythromycin can be pumped out by ABC transporter MsrA and MFS transporter Mef in *S. aureus*^46,47^. Our tested prenylated isoflavonoids did not show any erythromycin potentiation in *norA* overexpressing strain. Therefore, MsrA and/or Mef appear not to be the main targets of our tested compounds. Based on the notable potentiation observed with fluoroquinolones as verified NorA substrates, we hypothesize that NorA is the main target of these prenylated isoflavonoids.

With our findings, we postulate that glabrene and neobavaisoflavone mainly act as efflux pump inhibitors. In terms of potentiating fluoroquinolones, neobavaisoflavone and glabrene demonstrated promising activities in comparison with previously reported phytochemicals. Both neobavaisoflavone and glabrene reduced the effectiveness of ciprofloxacin by eightfold at concentrations of 19 and 39 µM, respectively, while non-prenylated isoflavonoids genistein and biochanin A gave the same fold reduction at concentrations of 222 µM and 105 µM, respectively^45^. Osthol, a prenylated coumarin, potentiated ciprofloxacin by fourfold at 25 µM, whereas sophoraflavanone G, a prenylated flavanone, was reported to potentiate norfloxacin by 16-fold at ¼ MIC (2.4 µM)^26,27^. Neobavaisoflavone showed similar potentiation of fluoroquinolone activity with the known NorA EPI reserpine at 10 µM, and eightfold reduction was observed at two times concentration.

Neobavaisoflavone and glabrene also showed antimicrobial activities against *S. aureus*. According to a previous classification of antimicrobial activities of natural compounds, neobavaisoflavone can be classified as a very good antibacterial against all three *S. aureus* strains (MIC ≤ 15 µg/mL), whereas glabrene can be classified as very good to good antibacterial (12.5 < MIC < 25 µg/mL)^30^. We postulate that glabrene and neobavaisoflavone potentially have dual mechanisms of action by acting as EPIs and antibacterials at specific concentrations.

### Glabrene and neobavaisoflavone did not show permeabilization and cytotoxicity at the tested EPI concentrations

We evaluated the secondary effect of the two most active compounds as membrane permeabilizers and their cytotoxicity properties. By performing the PI assay, we infer that the increase of fluorescence in the Eth assay was mainly due to efflux inhibition rather than membrane permeabilization. Yet, we should take into account that some prenylated isoflavonoids are good permeabilizers when they reach their MIC values^48^. The PI uptake is also facilitated when the cells have elevated PMF^40^. One putative explanation for some low levels of PI uptake indicated with neobavaisoflavone and glabrene is that by inhibiting the NorA efflux pump, less PMF is consumed and might have an effect on the steady-state PMF level. In other words, these two compounds might not have a permeabilization effect, but by inhibiting the EP, they elevate the PMF steady-state level, leading to enhanced PI uptake.

In addition to the PI assay, we assessed the cytotoxicity effect of glabrene and neobavaisoflavone in Caco-2 cell lines. According to our cytotoxicity assessment, glabrene and neobavaisoflavone did not show notable cytotoxic effects at a concentration up to 25 µg/mL (78 µM) and 12.5 µg/mL (39 µM), respectively. Despite that no IC_50_ was determined, the cytotoxicity of glabrene and neobavaisoflavone were lower compared to the cytotoxicity of the known NorA EPI reserpine (IC_50_ 30.07 ± 7.57 μM against HCT116 (p53+/+) colon cancer cells)^49^. One of the disadvantages of the Caco-2 cell lines is that, when compared to normal intestinal enterocytes, these cell lines have absorptive enterocytes only and no other epithelial cells, such as goblet cells and enteroendocrine cells^50^. Moreover, Caco-2 cell lines have different expressions of metabolic enzymes, leading to unrepresentative situations compared to normal intestinal cells^51^. To further evaluate the cytotoxicity properties of the compounds, experiments in a more representative model of normal intestinal cells should be performed, for example, primary cell lines, co-cultures (Caco-2 in combination with HT29-I cells), and intestinal organoids^50,52,53^.

### Predicted key molecular properties of prenylated isoflavonoids as potential NorA EPIs

**Figure 8:**
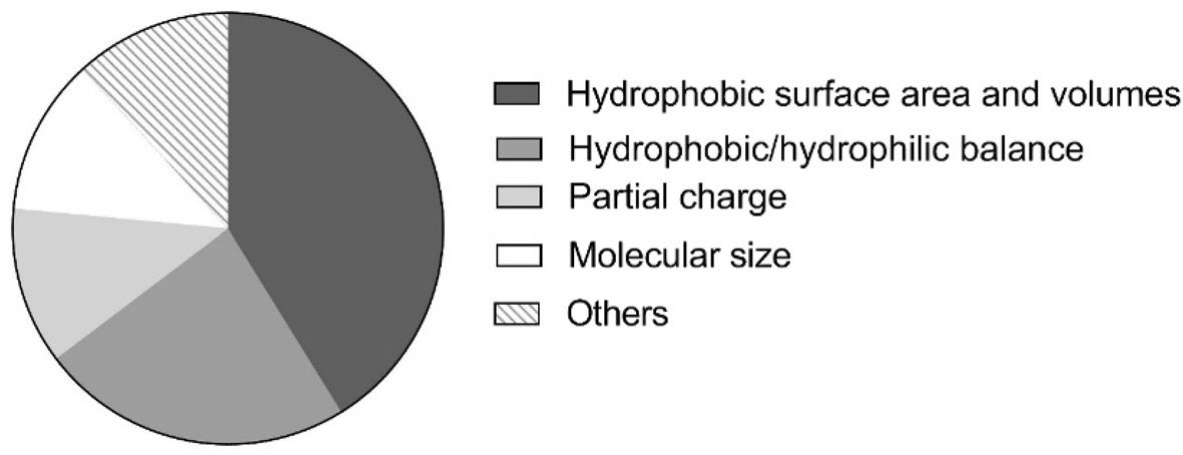
Molecular properties of prenylated isoflavonoids highly correlated (*r* > 0.95,* p* < 0.05) with EPI activities (fourfold reduction of ciprofloxacin MIC.

Based on the antibiotic potentiation activity (fourfold reduction of ciprofloxacin MIC), some molecular properties were found highly-correlated (*r* ≥ 0.95) with EPI activities of glabrene, neobavaisoflavone, glyceollin I, and glyceollin III (Fig. 8, Table S2). Hydrophobic surface area and volume-related descriptors (together 41%) and hydrophobic/hydrophilic balance descriptors (24%) accounted for the majority of descriptors highly-correlated with EPI activity. Partial charge-related descriptors (12%) and molecular size (12%) followed in importance. These results agree with our previous quantitative structure–activity relationships (QSAR) study where hydrophobicity, the balance of hydrophobicity/hydrophilicity, and (partial) charge were involved in the anti-MRSA (methicillin resistant *S. aureus*) activity of a larger set of prenylated isoflavonoids^54^. It seems that the same key molecular properties influence antibacterial activity and NorA EPI activity. Nonetheless, as noticed in our initial screening (Fig. 2B and Table S1), not all good NorA EPI candidates showed good antibacterial activity (and vice versa).

Hydrophobicity has been proposed to facilitate the interaction of prenylated isoflavonoids with bacterial membranes, whereas hydroxyl groups help keeping the hydrophobic/hydrophilic balance and facilitate intermolecular hydrogen bonds^54,55^. The presence of phenylalanine residues in the substrate binding pocket of NorA confers hydrophobicity to this site and may offer π–π stacking possibilities between aromatic rings of prenylated isoflavonoids and these residues^56^. In the future, a larger set of prenylated isoflavonoids is required to understand the structure–activity relationships of prenylated isoflavonoids as NorA EPIs. Furthermore, protein–ligand binding assays and co-crystallization of NorA with prenylated isoflavonoids will provide definitive confirmation of their binding and shed light into their mechanism of action as EPIs.

## Conclusions

The interplay of prenylated isoflavonoids as natural antimicrobials and efflux pump inhibitors against fluoroquinolone-resistant *S. aureus* makes these compounds interesting hits to develop new and potent RMAs. At concentrations up to ½ MIC in the *norA* overexpressing strain, glabrene and neobavaisoflavone showed fold changes in Eth accumulation of 5.9 and 4.5 times higher than the control, respectively, indicating an effective reduction of efflux pump activity. In addition, both compounds pronouncedly potentiated fluoroquinolones at a low concentration in the *norA* overexpressing *S. aureus* strain, resulting in an up to eightfold reduction of ciprofloxacin and norfloxacin MIC. This potentiation was not observed in the *norA* knockout strain. Neobavaisoflavone demonstrated a better activity than reserpine by showing higher Eth accumulation and less cytotoxicity. Moreover, neither notable membrane permeabilization nor cytotoxic effects of glabrene and neobavaisoflavone were observed at sub-inhibitory concentrations up to ½ MIC. These findings suggest that the prenylated isoflavonoids glabrene and neobavaisoflavone are potential NorA EPIs and have promising activities for use in combination therapy to tackle NorA-mediated fluoroquinolone resistance in *S. aureus.* Establishing the (quantitative) structure–activity relationships of prenylated isoflavonoids as NorA EPIs and elucidating their molecular mechanism of inhibition are essential steps for follow up studies.

## Methods

### Bacterial strains

*S. aureus* (SA)-1199B (a fluoroquinolone-resistant strain overexpressing *norA*), SA-1199 (Wild-type clinical isolate strain), and SA-K1758 (*norA* knockout strain) from BEI Resources were kindly provided by Prof. Michael J. Rybak (Wayne State University, USA) and Prof. Glenn W. Kaatz (Wayne State University, USA). The *norA* knockout strain (SA-K1758) is a derivative of *S. aureus* NCTC 8325-4, in which the *norA* gene was deleted and replaced with an *erm* cassette carrying the erythromycin resistance gene^23,36^. The bacteria were stored in a glycerol stock at − 80 °C.

### Media, reagents, and phytochemicals

Ethidium bromide (EtBr), propidium iodide (PI), phosphate buffer saline (pH 7.4), reserpine, erythromycin, and ciprofloxacin were purchased from Sigma Aldrich (St. Louis, MO, USA). Peptone physiological salt solution (PPS) was purchased from Tritium Microbiologie (Eindhoven, The Netherlands). Tryptone soy agar (TSA) and tryptone soy broth (TSB) were purchased from Oxoid Ltd (Basingstoke, UK). Dimethyl sulfoxide (DMSO) was purchased from Brunschwig Chemie B.V. (Amsterdam, The Netherlands) and ethyl acetate absolute was from Biosolve B.V. (Valkenswaard, The Netherlands). Neobavaisoflavone (≥ 95% purity) was supplied by PhytoLab GmbH & Co.KG (Vestenbergsgreuth, Germany). Glabridin (97% purity) was purchased from Wako (Osaka, Japan). Glabrene, glyceollin I, glyceollin III, glyceollin IV, glyceollidin II, dehydroglyceollin I, dehydroglyceollin III, dehydroglyceollin IV, and dehydroglyceollidin II were previously purified from roots of *Glycyrrhiza glabra* and from soybeans (Glycine max (L.) Merrill)^57,58^. All the tested phytochemicals have ≥ 90% purity.

### Antimicrobial susceptibility test

The minimum inhibitory concentrations (MIC) and minimum bactericidal concentrations (MBC) of compounds were determined by using broth microdilution method. Bacteria were streaked from − 80 °C glycerol stock onto TSA agar plates and incubated at 37 °C for 24 h. After overnight incubation, one colony was transferred in 10 mL TSB and grown for another 24 h at the same temperature. Afterwards, the inoculum was diluted in TSB to reach an average inoculum of 5.31 ± 0.16, 5.33 ± 0.22, 5.04 ± 0.21 log_10_ colony forming units (CFU)/mL for SA-1199B, SA-1199, and SA-K1758, respectively. Stock solutions of compounds were prepared in DMSO and subsequently diluted in TSB. Antibiotics were prepared in phosphate-buffered saline (PBS) (0.1% v/v acetic acid). Next, tested compounds (100 µL) and inoculum (100 µL) were mixed in a 96-well plate, where the final concentration of compounds ranged from 6.25 to 50 µg/mL (solvent concentration was at max. 2% v/v). The negative control (inoculum in TSB with 2% DMSO) and blank (TSB medium without bacteria) were included in each assay. Microbial growth was measured spectrophotometrically every 10 min at OD_600_ (Tecan-Infinite 200 Pro M nanoplate reader) at 37 °C for 24 h under continuous shaking.

When there was no change in OD_600_ (flat line) after 24 h measurement, cell viability was measured by plate counting to obtain the MIC and MBC values^48^. The MIC was defined as the lowest concentration of compound (drug) that resulted in equal or a lower cell count compared to the initial inoculum. MBC was defined as the lowest concentration of compound that showed more than 99.9% bacterial killing from the initial inoculum^59^. The activities of prenylated isoflavonoids against SA-1199B, SA-1199, and SA-K1758 were tested in technical triplicates with at least two biological repetitions each.

### Ethidium accumulation assay

Ethidium accumulation assays were performed according to Coldham et al. with a slight modification^60^. One colony from an overnight TSA plate was grown in 3 mL TSB medium for approximately 4 h (shaking 200 rpm, at 37 °C) until reaching an OD_600_ 1.2–1.4. The inoculum was aliquoted into 1 mL microtubes and centrifuged at 4000 g for 5 min. The supernatant was discarded, and the cell pellet washed once with PBS buffer (pH 7.4), supplemented with 1 mM MgSO_4_. The cell pellet was resuspended in the same buffer at OD_600_ = 0.6. The average inoculum sizes were 9.19 ± 0.17, 9.22 ± 0.14, and 9.00 ± 0.10 log10 CFU/mL for SA-1199B, SA-1199 and SA-K1758, respectively.

Compound stock solutions were prepared in 100% DMSO and subsequently PBS was used to dissolve the compounds and EtBr. The cell suspension (150 μL) was placed into a black 96-well plate with 10 μL EtBr (final concentration 3 μg/mL or 7.6 µM) and 40 μL of compounds (max. 2% v/v DMSO). Negative control (cells with 2% DMSO and EtBr) and blanks (compounds with both buffer and EtBr) were prepared for each assay. The addition of reserpine (20 µg/mL) was used as a positive control^21^. The ethidium fluorescence was measured by using Spectramax ID3 for 60 min (37 °C), with excitation 520 nm and emission 620 nm. Blanks (each tested compound in buffer with and without EtBr) were measured for each sample to check and correct background fluorescence. The fold change in Eth accumulation was calculated by taking the final fluorescence unit (RFU) relative to the negative control at t = 60 min, as described by Šimunović et al.^61^.

1 $$Fold \, change \, \left(FC\right)=\frac{RFU \, of \, cells \, treated \, with \, EPI}{RFU \, of \, negative \, control}$$

The first screening with EPI candidates was performed in *norA* overexpressing strain (SA-1199B) with concentrations ranging from 6.25 to 50 µg/mL. The fold changes in Eth accumulation were shown as means with standard deviation. Statistical significance was analyzed by a student’s t-test with Welch’s correction^35^. All samples were measured in technical triplicates with two biological repetitions each.

### Antibiotic potentiation study (checkerboard assay)

The antibiotic potentiation effect was assessed by using the same procedure as described in the antimicrobial susceptibility test. Bacteria were diluted in TSB to obtain an initial inoculum of 5.37 ± 0.13 and 5.10 ± 0.01 log10 CFU/mL for SA-1199B and SA-K1758, respectively. The 96-well plate was filled with two-fold serial dilution of the antibiotics in combination with prenylated isoflavonoids, starting from ½ to 1/8 MIC. For those compounds with MIC values higher than 50 µg/mL, such as the pterocarpans (Fig. 1), a series of 20, 30, 40, and 50 µg/mL was used. In a 96-well plate, 50 µL of antibiotics and prenylated isoflavonoids were mixed with 100 µL of inoculum (max. 2% DMSO and 0.01% v/v acetic acid). All experiments were done in technical duplicates with at least two biological repetitions each. The fractional inhibitory concentration index (FICI) was calculated according to the formula below:

2 $$FICI= \frac{MIC \, of \, phytochemical \, in \, combination}{MIC \, of \, \, phytochemical \, alone}+ \frac{MIC \, of \, antibiotic \, in \, combination}{MIC \, of \, antibiotic \, alone}$$

FICI ≤ 0.5 was defined as “synergy”; 0.5 < FICI < 1 was considered as “partial synergy”; FICI = 1 was categorized as additive; 1 < FICI < 4 was observed as “indifferent” and FICI ≥ 4.0 was indicated as “antagonistic”^62^. In case the prenylated isoflavonoids did not exhibit an antimicrobial effect itself (no detectable MIC at the maximum concentration tested), the FICI was estimated with the highest concentration of the compound examined.

### Membrane permeability assay

Propidium iodide (PI) was used to assess membrane permeabilization, as previously reported with slight modifications^48^. In brief, bacteria were grown on TSA plates incubated overnight at 37 °C. One colony was transferred in 25 mL TSB and cells were grown for 20 h at 37 °C. The cell cultures were harvested by centrifugation at 4,696 g, 4 °C for 5 min. The cell pellet was washed twice with 5 mL PPS. Subsequently, the pellet was resuspended in 5 mL of PPS to obtain average inoculum sizes of 9.75 ± 0.01, 9.74 ± 0.35 and 9.30 ± 0.06 log10 CFU/mL for SA-1199B, SA-1199, and SA-K1758, respectively. The inoculum (100 μL) and tested compounds (50 µL) at final concentrations of up to ½ MIC, were added to each well in a black 96-well plate with a transparent bottom. A positive control was prepared by heating the cells for 10 min at 95 °C. The negative control contained cells and PI with a final volume of 200 µL. Fluorescence of the initial samples (tested compounds with PI; PI only, without compounds) were checked for the intrinsic fluorescence of prenylated isoflavonoids. Experiments were performed at least in technical duplicates with two biological repetitions each. The relative PI uptake was calculated by taking the final fluorescence unit (RFU) relative to the negative control at t = 120 min.

3 $$Relative \,PI \,uptake =\frac{RFU \,of \,cells \,treated \,with \,compound}{RFU \,of \,negative \,control}$$

### Cytotoxicity assay

Following 4- and 24-h incubation periods, the cytotoxic effects of glabrene and neobavaisoflavone were tested at a range of concentrations starting from ½ to 2 × MIC. The cytotoxic effects on human colonic carcinoma cell line Caco-2 were assessed by measuring intracellular lactate dehydrogenase (LDH) leakage in the supernatant and analyzing the results using an LDH cytotoxicity detection kit (Roche Applied Science, Almere, The Netherlands)^63^. Compounds were dissolved in DMSO and the maximum DMSO concentration used was 1.56% (*v/v*), which reportedly did not affect cell cytotoxicity. LDH activity in the supernatant was expressed as a percentage of the maximum releasable LDH in cells (Caco-2 cells treated with 1% Triton X-100) and calculated with Eq. (4).

4 $$Cytotoxicity \left(\%\right)= \frac{exp. value - low \, control}{high \, control - low \, control} \times 100$$

Here, *exp.value* is UV absorbance at 492 nm (Spectramax M2, Molecular Devices). *Low control* is the spontaneous LDH release in untreated cells (Caco-2 cells in culture medium), and *high control* is the maximum releasable LDH in cells (Caco-2 cells treated with 1% Triton X-100). The compounds were considered cytotoxic when > 20% cytotoxicity was observed.

Effects on cell viability in Caco-2 cells were assessed after incubation of 4 h. The Caco-2 cell viability was evaluated by measuring the cleavage of the tetrazolium salt WST-1 to formazan by cellular mitochondrial dehydrogenases. The cell viability was examined by using a WST-1 cell viability kit (PromoKine, Heidelberg, Germany), as per the manufacturer’s instructions. Cell viability was expressed as percentage of the control cells (Caco-2 cells in culture medium) and calculated with Eq. (5).

5 $$Viability \left(\%\right)= \frac{exp. value}{low \, control} \times 100$$

Here, *exp.value* is UV absorbance at 450 nm (Multiskan Ascent, Thermo Fisher Scientific), and *low control* is spontaneous cleavage of WST-1 to formazan by mitochondrial dehydrogenases in untreated cells. All data were presented in GraphPad Prism version 9.

### Prediction of molecular properties

The 3D optimized structures of prenylated isoflavonoids and molecular properties (descriptors) were calculated using Molecular Operating Environment (MOE) software (version 2019.0102, Chemical Computing Group) after MMFF94x energy minimization (gradient 0.01). Correlation between molecular descriptors and antibiotic potentiation activity (i.e. concentration required for fourfold reduction of ciprofloxacin MIC) was assessed based on the Pearson correlation coefficient (*r*). List of highly significantly correlated (*r* ≥ 0.95, *p* < 0.05) molecular descriptors is in Table S2 in the supplementary file.

### Supplementary Information

Supplementary Information.

## Data Availability

Upon reasonable request, the corresponding author will provide the datasets used and/or analyzed during the study.

## Abbreviations

EPIs: Efflux pump inhibitors
AMR: Antimicrobial resistance
RMAs: Resistance-modifying agents
MFS: Major facilitator superfamily
5′-MHC: 5′-Methoxy-hydnocarpin
Eth: Ethidium
EtBr: Ethidium bromide
PI: Propidium iodide
MIC: Minimum inhibitory concentration
MBC: Minimum bactericidal concentrations
FICI: Fractional inhibitory concentration index
CFU: Colony forming units
RFU: Relative fluorescence unit
PMF: Proton-motive force
PPS: Peptone physiological salt solution
TSA: Tryptone soy agar
TSB: Tryptone soy broth
DMSO: Dimethyl sulfoxide
LDH: Lactate dehydrogenase
MOE: Molecular operating environment
CCCP: Cyanide-m-chlorophenyl hydrazone
QSAR: Quantitative structure–activity relationships
