# Supplementary material for: Prenylated isoflavonoids from Fabaceae against the NorA efflux pump in Staphylococcus aureus
Source: Sci Rep. 2023 Dec 18;13:22548. doi: 10.1038/s41598-023-48992-8 (PMC10728173; doi:10.1038/s41598-023-48992-8)
Supplement: Supplementary file 1 — Supplementary Information. [file 41598_2023_48992_MOESM1_ESM.docx]

**Prenylated isoflavonoids from Fabaceae** **against the NorA efflux pump in *Staphylococcus aureus***

Marina Ika Irianti^a,b^, Jean-Paul Vincken^a^, Sarah van Dinteren^a^, Ellen ter Beest^a^, Klaas Martinus Pos^c^, Carla Araya-Cloutier^a*^

*^a^Laboratory of Food Chemistry, Wageningen University and Research, Bornse Weilanden 9, 6708 WG, Wageningen, The Netherlands*

*^b^ Laboratory of Microbiology and Biotechnology, Faculty of Pharmacy, Universitas Indonesia, 16424, Depok, Indonesia*

*^c^Institute of Biochemistry, Goethe-University Frankfurt, Frankfurt am Main D-60438, Germany*

*Corresponding author. Address**:** Laboratory of Food Chemistry, Wageningen University and Research, Bornse Weilanden 9,6708 WG, Wageningen, The Netherlands.

E-mail address: [carla.arayacloutier@wur.nl](mailto:carla.arayacloutier@wur.nl)

**Supporting Information**

**Table S 1** MIC determination of prenylated isoflavonoids in SA-1199B; N. D. = not determined

| **No** | **Compound** | **MIC (µg/mL)** | **Cell number**  **(CFU/mL)** |
| --- | --- | --- | --- |
|  | **Isoflavan** |  |  |
| 1 | Glabridin | 6.25<MIC<12.5 | N. D. |
|  | **Isoflavene** |  |  |
| 2 | Glabrene | 25 | Log 3.53 ± 2.33 |
|  | **Isoflavone** |  |  |
| 3 | Neobavaisoflavone | 12.5 | Log 5.33 ± 0.46 |
|  | **6a-OH-Pterocarpan** |  |  |
| 4 | Glyceollidin II | >50* | N. D. |
| 5 | Glyceollin I | 80 | Log 3.08 ± 0.14 |
| 6 | Glyceollin III | 80 | Log 4.67 ± 0.47 |
| 7 | Glyceollin IV | 25 | Log 5.43 ± 0.07 |
|  | **6a,11a-Pterocarpene** |  |  |
| 8 | Dehydroglyceollin I | 12.5 | Log 3.02 ± 0 |
| 9 | Dehydroglyceollin III | 50 | Log 4.93 ± 0.67 |
| 10 | Dehydroglyceollin IV | 6.25 | Log 1.69 ± 0.12 |
| 11 | Dehydroglyceollidin II | 6.25 | Log 5.95 ± 0 |

*Growth inhibition was indicated through 24 hr measurement and MIC value was slightly higher than 50 µg/mL.

**Table S 2** Molecular descriptors highly correlated (*r* ≥ 0.95, *p <* 0.05) with antibiotic potentiation of prenylated isoflavonoids (based on the concentration required for 4-fold reduction of ciprofloxacin MIC).

| **No.** | **Descriptors with R_pearson_ >0.95 and R_pearson_<-0.95** | **Values** | **Definition** | **Category** |
| --- | --- | --- | --- | --- |
| 1 | ASA_H | 0.951631212 | Water accessible surface area of all hydrophobic (\|qi \|<0.2) atoms | Hydrophobic Surface area and volumes |
| 2 | vsurf_A | 0.953833163 | Amphiphilic moment | Hydrophobic/  hidrophilic balance |
| 3 | dens | 0.95643452 | Mass density: molecular weight divided by van der Waals volume. | Molecular size |
| 4 | PEOE_PC+ | 0.95977028 | Relative positive partial charge: the largest positive qi divided by the sum of the positive qi | Partial charge |
| 5 | PEOE_PC- | -0.95977028 | Total negative partial charge: the sum of the negative qi. | Partial charge |
| 6 | vsurf_D1 | -0.98319279 | Hydrophobic volume at -0.2 kcal/mol. | Hydrophobic surface area and volumes |
| 7 | vsurf_D2 | -0.97646076 | Hydrophobic volume at -0.4 kcal/mol. | Hydrophobic surface area and volumes |
| 8 | vsurf_D3 | -0.9656309 | Hydrophobic volume at -0.6 kcal/mol | Hydrophobic surface area and volumes |
| 9 | vsurf_D4 | -0.97788872 | Hydrophobic volume at -0.8 kcal/mol. | Hydrophobic surface area and volumes |
| 10 | vsurf_D5 | -0.96493477 | Hydrophobic volume at -1.0 kcal/mol. | Hydrophobic surface area and volumes |
| 11 | vsurf_D8 | -0.98099145 | Hydrophobic volume at -1.6 kcal/mol. | Hydrophobic surface area and volumes |
| 12 | vsurf_HB7 | 0.972111718 | H-bond donor capacity at -5.0 kcal/mol | Others |
| 13 | vsurf_ID6 | 0.976580256 | Hydrophobic integy moment at -1.2 kcal/mol | Hydrophobic/  hidrophilic balance |
| 14 | vsurf_IW2 | 0.983699465 | Hydrophilic integy moment at -0.5 kcal/mol. | Hydrophobic/  hidrophilic balance |
| 15 | vsurf_IW7 | 0.992160587 | Hydrophilic integy moment at -5.0 kcal/mol. | Hydrophobic/  hidrophilic balance |
| 16 | vsurf_W7 | 0.972111718 | Hydrophilic volume at -5.0 kcal/mol. | Others |
| 17 | Weight | 0.972111718 | Molecular weight | Molecular size |

| **A**   | **B**   |
| --- | --- |
| **C**   | **D**   |

**Figure S 1.** Eth accumulation in the absence or presence of neobavaisoflavone (A), glabrene (B), glyceollin I (C), glyceollin III (D) at the indicated concentrations in the *norA* overexpressing strain SA-1199B. The data were plotted from two biological repetitions with standard deviations.

| **A**   | **B**   |
| --- | --- |
| **C**   | **D**   |

**Figure S 2.** Eth accumulation in the absence or presence of neobavaisoflavone (A), glabrene (B), glyceollin I (C), glyceollin III (D) at the indicated concentrations in the wildtype strain SA-1199. The data were plotted from two biological repetitions with standard deviations.

| **A**   | **B**   |
| --- | --- |
| **C**   | **D**   |
| **Figure S 3.** Eth accumulation in the absence or presence of neobavaisoflavone (A), glabrene (B), glyceollin I (C), glyceollin III (D) at the indicated concentrations in the *norA* knockout strain SA-K1758. The data were plotted from two biological repetitions with standard deviations.      **Figure S 4.** Fluorescence measurement of the blanks: (A) prenylated isoflavonoids in the presence of EtBr (7.6 µM) and PBS buffer and (B) prenylated isoflavonoids in PBS buffer. The fluorescence was measured for 60 min (ex: 520 nm, em: 620 nm). | |

**Figure S 5.** Propidium iodide (PI) assay in the presence of prenylated isoflavonoids at ½ MIC, reserpine or proton motive force uncoupler CCCP. Cells were prepared similarly to membrane permeability assay with a slight modification. After the washing steps, cells were preincubated with 40 µM CCCP on ice for 10 min, followed by two wash steps with PPS without CCCP. Ns denotes non-significant (*p* > 0.05) relative to negative control, which contained cells treated with CCCP but without addition of the modulators. The positive control was cells treated with CCCP that are heated at 95 °C for 10 min.
